# Suicidal ideation, suicide plans and suicide attempts among Australian adults: Findings from the 2020–2022 National Study of Mental Health and Wellbeing

**DOI:** 10.1177/00048674241256753

**Published:** 2024-06-10

**Authors:** Vikas Arya, Philip Burgess, Sandra Diminic, Meredith G Harris, Tim Slade, Matthew Sunderland, Caley Tapp, Joshua Vescovi, Jane Pirkis

**Affiliations:** 1 Centre for Mental Health and Community Wellbeing, Melbourne School of Population and Global Health, The University of Melbourne, Melbourne, VIC, Australia; 2 School of Public Health, The University of Queensland, Brisbane, QLD, Australia; 3 Queensland Centre for Mental Health Research, Wacol, QLD, Australia; 4 The Matilda Centre for Research in Mental Health and Substance Use, The University of Sydney, Sydney, NSW, Australia

**Keywords:** Suicidal ideation, suicide plans, suicide attempts, 2020–2022 National Study of Mental Health and Wellbeing, self-harm without suicidal intent

## Abstract

**Objective:**

This study aimed to describe the epidemiology of suicidal ideation, suicide plans and suicide attempts, examine services received for suicide attempts, and explore the relationship between suicide attempts and self-harm without suicidal intent.

**Methods:**

We used survey data from the 2020–2022 National Study of Mental Health and Wellbeing, which involved a nationally representative sample of Australian adults aged 16–85 (*n* = 15,893). Comparisons were made with the 2007 National Study of Mental Health and Wellbeing (*n* = 8841).

**Results:**

In 2020–2022, the proportions of adults who had experienced suicidal ideation, suicide plans and suicide attempts during their lifetime were 16.6%, 7.5% and 4.9%, respectively. The proportions who had experienced these in the past 12 months were 3.3%, 1.1% and 0.3%. The odds of experiencing suicidal ideation and making a suicide plan were significantly higher in 2020–2022 than in 2007. Groups at heightened risk of suicidal ideation, suicide plans and/or suicide attempts in the previous 12 months were males, young people, people who were gay, lesbian, or bisexual or used some other term to describe their sexual identity, people outside the labour force, people from disadvantaged areas and people with mental disorders. Two-fifths of those who attempted suicide during the previous 12 months did not use health services following their attempt, and two-thirds also self-harmed without suicidal intent.

**Conclusion:**

The implications of these findings for the forthcoming National Suicide Prevention Strategy are discussed. Suicidal thoughts and behaviours confer risk for suicide and are significant problems in their own right. Their prevention requires a strong whole-of-government response.

## Introduction

Suicide is a major public health problem. The Australian Bureau of Statistics (ABS) (2023a) reported 3249 suicides in Australia in 2022, or 12.5 per 100,000 population. Although suicides are reasonably accurately counted in Australia (Snowdon and Choi, 2020), non-fatal suicidal behaviours and suicidal ideation are not. This is important because suicidal ideation, suicide plans and suicide attempts are often precursors to suicide (Nock et al., 2008; Turecki et al., 2019).

The need for better surveillance of suicidal thoughts and behaviours has increasingly been recognised in Australia (National Suicide Prevention Adviser, 2020). The National Suicide and Self-harm Monitoring System (NSSHMS) (Australian Institute of Health and Welfare, 2023) allows rates of self-harm to be tracked over time, albeit with some limitations. Episodes of self-harm are only counted if they involve hospital admissions or ambulance attendances, which underestimates rates in the community. The unit of counting is the event, making it difficult to determine the number of individuals who self-harm annually. It is not always possible to distinguish between self-harm with suicidal intent (i.e. a suicide attempt) and self-harm for other reasons (e.g. emotional relief). These limitations can only be overcome through population-based surveys.

The 2020–2022 National Study of Mental Health and Wellbeing (NSMHWB) provides the opportunity to estimate community-based rates of suicidal thoughts and behaviours (ABS, 2023c). Previous versions of the survey, conducted in 1997 and 2007, were used to make similar estimates (Johnston et al., 2009; Pirkis et al., 2000). The current study only makes comparisons with the 2007 estimates for two reasons. The first of these is that changes over the past 15 years are more relevant and meaningful than long-term changes. The second is that the 2020–2022 survey, like the 2007 survey, used the World Mental Health Composite International Diagnostic Interview (WMH-CIDI) version 3.0, whereas the 1997 survey used the WMH-CIDI version 2.1. These WMH-CIDI versions differed in the way they measured and classified mental disorders, using different algorithms and sequencing among other variations (ABS, 2023b). In addition to these time-based comparisons, current study also examines services received for suicide attempts in 2020–2022, and the relationship between suicide attempts and self-harm without suicidal intent in the same period. The study is timely because a new National Suicide Prevention Strategy is due for release in 2024 (National Suicide Prevention Office, 2022).

## Method

### Data source

The 2020–2022 NSMHWB was a cross-sectional survey conducted by the ABS which involved a household sampling strategy. The original plan was to recruit a single sample of Australian adults aged 16–85, but due to the COVID-19 pandemic, two separate cohorts were recruited (one between 5 December 2020 and 31 July 2021, and the other between 4 December 2021 and 31 October 2022). The ABS (2023b) has since created a combined sample with appropriate weights. We used the combined data in the current study because the greater sample size allowed for more precise estimates and meant that fewer data points were suppressed.

### Sampling strategy and participant selection

Participants aged 16–85 were selected for each cohort via a two-stage sampling strategy. Households were randomly selected, and household composition information was obtained. One person aged 16–85 years was randomly selected from each household and invited to complete a face-to-face survey administered as an interview by a trained interviewer. Individuals aged 16–24 had a higher selection probability compared to other age groups to improve estimates for this group. The study sampled participants from all states/territories and from both urban and rural areas (ABS, 2023c). The survey was broadly representative of the national Australian population aged 16–85 years.

The final sample consisted of 15,893 participants (7409 males and 8482 females; 5554 from the first cohort and 10,339 from the second), with a response rate of 52.0%.

### The survey instrument

The 2020–2022 survey asked about mental disorders and suicidal thoughts and behaviours. The World Mental Health Survey Initiative version of the Composite International Diagnostic Interview version 3.0 (WMH-CIDI 3.0) was used as the basis for the survey. Additional socio-demographic, clinical and service use questions that were relevant to the Australian context were included.

In the current study, we focused on questions relating to suicidal thoughts and behaviours. Participants were asked whether they had had any suicidal thoughts (‘Have you ever seriously thought of taking your life?’), had made plans for suicide (‘Did you ever make a plan about how you would take your life?’) and had attempted suicide in their lifetime (‘Did you ever try to take your life?’). Those who responded affirmatively to each question were then asked whether they had experienced these thoughts and behaviours in the past 12 months (‘Did you seriously think about taking your life any time in the past 12 months?’; ‘Did you make a plan about how you would take your life at any time in the past 12 months?’; and ‘Did you try to take your life in the past 12 months?’). If a person did not indicate that they had seriously thought about taking their own life during their lifetime or in the past 12 months, they were not asked about suicide plans or attempts over the same period.

Participants who indicated that they had attempted suicide in the past 12 months were asked ‘Did you get medical attention or mental health care from any of the following services or health professionals immediately following your attempt to take your own life?’ and given a series of options. If they endorsed any of these, they were then asked whether they received the services they needed from the given service or professional. Those who had made more than one attempt during the past 12 months were asked to consider the most recent attempt.

We also used a set of questions that considered self-harm without suicidal intent. Participants were asked whether they had self-harmed in their lifetime (‘Have you ever intentionally harmed yourself in any way, but not with the intention of taking your life?’) and in the past 12 months (‘Have you intentionally harmed yourself in the last 12 months?’). Again, the 12-month question relied on participants having responded in the affirmative to the lifetime question.

We used participants’ responses to questions about their sex and age and additional socio-demographic questions. These were Indigenous status, country of birth, sexual orientation and employment status. In addition, we used pre-coded information on the remoteness and socio-economic status of the area in which they lived. Remoteness was based on the Accessibility/Remoteness Index of Australia (ARIA), which classifies remoteness according to relative access to services (Australian Centre for Housing Research, 2023). Socio-economic status was based on the Index of Relative Socioeconomic Disadvantage (IRSD) of the Socioeconomic Indexes for Areas (SEIFA) (ABS, 2023d). We also used information on mental disorders based on an algorithm that was applied to responses to certain clinical questions to determine whether participants met the criteria for 12-month affective, anxiety and substance use disorders according to the *Diagnostic and Statistical Manual of Mental Disorders* (4th ed.; *DSM*-IV; American Psychiatric Association, 2000).

### Comparability with the 2007 NSMHWB

The 2020–2022 survey was broadly comparable with the 2007 survey (ABS, 2023c; Slade et al., 2009). The sampling, recruitment and survey administration processes was similar. The 2007 sample comprised 8841 adults of the same age range. The 2007 survey had the WMH CIDI at its core. It asked comparable questions about suicidal thoughts and behaviours, although some of the terminology was updated to reflect current thinking. In particular, the phrase ‘commit suicide’ from 2007 was replaced with the phrase ‘take your life’. The former term is no longer preferred because it has connotations of crime and sin (Beaton et al., 2013). The 2007 survey did not include questions about services received immediately following a suicide attempt or questions about self-harm without suicidal intent.

### Data analysis

We used the ‘Survey’ package within R software version 4.3.0 and conducted all analyses in the ABS’s secure DataLab environment (Lumley, 2023; R Core Team, 2023). Primary and secondary suppression rules were used for cell with counts of <10 to ensure that no individuals could be identified. All point estimates and standard errors were calculated using weights and replicate weights, respectively. 95% confidence intervals (CIs) were calculated using an Adjusted Wilson Score which accounted for weighting (Dean and Pagano, 2015).

Data were combined from the 2020–2022 and 2007 surveys (Moser et al., 2013). Lifetime and 12-month rates of suicidal ideation, suicide plans and suicide attempts, stratified by sex, were estimated for the 2020–2022 and 2007 surveys. Twelve-month rates of suicidal ideation, suicide plans and suicide attempts, stratified by age group, were also estimated for the two surveys; 12-month rates for suicide attempts for some age groups could not be estimated due to low counts.

The proportions of those who made a suicide attempt in the past 12 months who received services and the extent to which these services met their needs were gauged. Twelve-month rates of self-harm without suicidal intent and their association with suicide attempts were also estimated. The service receipt and non-suicidal self-harm figures were only estimated for the 2020–2022 survey; as noted above, there were no equivalent questions in the 2007 survey.

We used logistic regression to investigate the likelihood of having 12-month suicidal ideation, suicide plans and suicide attempts in the 2020–2022 survey compared to the 2007 survey, specifying the models by sex and age groups. Associations between the above socio-demographic and clinical factors and 12-month suicidal ideation, suicide plans and suicide attempts for the 2020–2022 survey were also investigated through multivariable logistic regression.

## Results

Table 1 shows that in the 2020–2022 survey, 16.7% of participants indicated that they had experienced suicidal ideation at some point during their lifetime, 7.5% reported making a suicide plan and 4.9% said they had attempted suicide. The equivalent figures from the 2007 survey were 13.3%, 4.0% and 3.2%. The 2020–2022 estimates equate to around 3.3 million Australian adults seriously considering ending their lives at some point in their lifetime, 1.5 million making a suicide plan and nearly a million attempting suicide.

**Table 1.** Lifetime and 12-month rates of suicidal ideation, suicide plans and suicide attempts, by sex, 2007 and 2020–2022.

		Males		Females		Persons	
		EPC (’000)	% [95% CI]	EPC (’000)	% [95% CI]	EPC (’000)	% [95% CI]
Lifetime rates							
2020–2022	Suicidal ideation	1466.4	15.0 [14.0, 16.1]	1,837.3	18.3 [16.9, 19.7]	3307.2	16.7 [15.8, 17.6]
	Suicide plans	659.4	6.8 [6.1, 7.5]	816.1	8.1 [7.1, 9.2]	1478.6	7.5 [6.8, 8.2]
	Suicide attempts	398.3	4.1 [3.6, 4.6]	569.2	5.7 [5.0, 6.4]	970.6	4.9 [4.5, 5.3]
2007	Suicidal ideation	914.7	11.5 [10.1, 13.1]	1207.6	15.0 [13.7, 16.3]	2122.4	13.3 [12.4, 14.2]
	Suicide plans	234.2	2.9 [2.3, 3.8]	398.6	4.9 [4.2, 5.8]	632.8	4.0 [3.4, 4.6]
	Suicide attempts	168.4	2.1 [1.6, 2.8]	351.9	4.4 [3.7, 5.1]	520.3	3.2 [2.9, 3.7]
12-month rates							
2020–2022	Suicidal ideation	295.8	3.0 [2.6, 3.6]	352.8	3.5 [3.0, 4.1]	648.7	3.3 [2.9, 3.7]
	Suicide plans	113.1	1.2 [0.9, 1.5]	113.6	1.1 [0.9, 1.5]	226.7	1.1 [0.9, 1.4]
	Suicide attempts	26.7	0.3 [0.2, 0.5]	30.7	0.3 [0.2, 0.4]	57.4	0.3 [0.2, 0.4]
2007	Suicidal ideation	146.7	1.8 [1.4, 2.5]	221.3	2.7 [2.2, 3.4]	368.1	2.3 [1.9, 2.7]
	Suicide plans	33.5	0.4 [0.3, 0.6]	57.5	0.7 [0.5, 1.1]	91.0	0.6 [0.4, 0.8]
	Suicide attempts	22.6	0.3 [0.1, 0.5]	42.7	0.5 [0.3, 0.8]	65.3	0.4 [0.3, 0.6]

EPC: estimated population count; 95% CI: 95% confidence interval.

Table 1 also shows the 12-month rates of suicidal ideation, suicide plans and suicide attempts for the 2020–2022 survey, again making comparisons with the 2007 survey. In 2020–2022, 3.3% of participants indicated that they had seriously thought about suicide in the past 12 months, 1.1% noted that they had made a suicide plan and 0.3% reported that they had attempted suicide. The corresponding figures from the 2007 survey were 2.3%, 0.6% and 0.4%, respectively. The 2020–2022 estimates equate to 648,700 Australian adults considering suicide in 2020–2022, 226,700 making a suicide plan and 57,400 attempting suicide.

**Figure 1.**
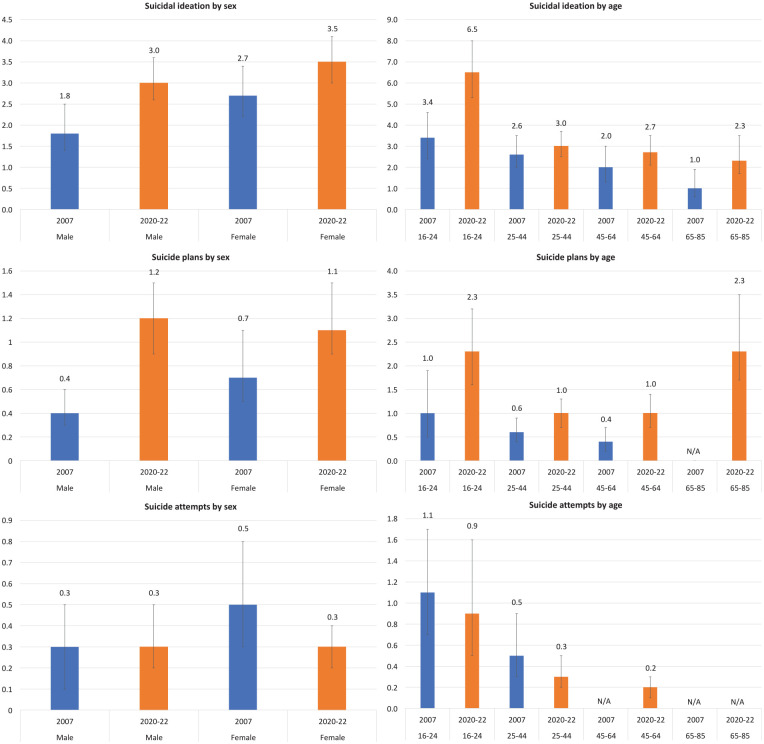
12-month rates of suicidal ideation, suicide plans and suicide attempts, by sex and age, 2007 and 2020–2022. N/A indicates that data are not available for publication due to small cell counts.

Figure 1 shows the rates of suicidal ideation, suicide plans and, where possible, suicide attempts identified in the 2007 and 2020–2022 surveys broken down by sex and age. It highlights increases in rates of suicidal ideation and suicide plans for males, and increases in rates of suicidal ideation for those aged 16–24. Table 2 provides more detail, comparing the relevant suicidal outcomes in 2007 and 2020–2022 through the logistic regression analysis. The odds of experiencing suicidal ideation and making a suicide plan were higher for the overall adult population in 2020–2022 than they were in 2007. Males had higher odds of reporting suicidal ideation and suicide plans in 2020–2022 than they did in 2007. The odds also increased over time for participants aged 16–24 for suicidal ideation and suicide plans, and, similarly, for participants aged 45+ for suicidal ideation and suicide plans.

**Table 2.** Odds of 12-month suicidal ideation, suicide plans and suicide attempts in 2020–2022 compared with 2007, by sex and age.

	Total sample		Males		Females		Aged 16–24		Aged 25–44		Aged 45+	
	OR	95% CI	OR	95% CI	OR	95% CI	OR	95% CI	OR	95% CI	OR	95% CI
Suicidal ideation	1.44	[1.15, 1.79]	1.66	[1.16, 2.37]	1.29	[0.98, 1.70]	2.19	[1.49, 3.20]	1.05	[0.73, 1.51]	1.58	[1.05, 2.38]
Suicide plans	2.02	[1.40, 2.93]	2.77	[1.69, 4.53]	1.59	[0.94, 2.70]	2.28	[1.08, 4.80]	1.53	[0.84, 2.79]	2.71	[1.52, 4.85]
Suicidal attempts	0.71	[0.42, 1.20]	0.96	[0.38, 2.45]	0.57	[0.31, 1.08]	0.90	[0.43, 1.90]	0.48	[0.19, 1.21]	1.46	[0.48, 4.48]

OR: odds ratio; 95% CI: 95% confidence interval.

Table 3 shows the socio-demographic and clinical correlates of 12-month suicidal ideation, suicidal plans and suicide attempts for the 2020–2022 survey. Females had lower odds than males of making a suicide plan. The youngest age group (16–24) had higher odds of attempting suicide compared to those aged 45+. Compared with heterosexual participants, those who indicated they were gay/lesbian/bisexual/different term had higher odds of experiencing suicidal ideation, making a suicide plan and attempting suicide. Participants who were not in the labour force had higher odds of experiencing suicidal ideation than their employed counterparts. Those living in the most disadvantaged areas had higher odds of attempting suicide than those living in the least disadvantaged areas. Participants with any affective disorder in the past 12 months had higher odds of experiencing suicidal ideation, making a suicide plan and attempting suicide than those without. Participants with any 12-month anxiety disorder also had higher odds of each of the three outcomes. The same was true for participants with any 12-month substance use disorder.

**Table 3.** Multivariable logistic regression of socio-demographic and clinical correlates of 12-month suicidal thoughts and behaviours, 2020–2022.

		Suicidal ideation	Suicide plans	Suicide attempts
		aOR [95% CI]	aOR [95% CI]	aOR [95% CI]
Sex	Males	1.00	1.00	1.00
	Females	0.80 [0.63, 1.03]	**0.66 [0.46, 0.94]**	0.70 [0.31, 1.59]
Age	16–24	1.31 [0.89, 1.92]	1.04 [0.60, 1.80]	**2.42 [1.01, 5.82]**
	25–44	0.98 [0.68, 1.40]	0.71 [0.46, 1.10]	1.13 [0.52, 2.46]
	45+	1.00	1.00	1.00
Indigenous status	Aboriginal or Torres Strait Islander	1.63 [0.82, 3.26]	2.38 [0.75, 7.62]	1.44 [0.16, 13.13]
	Neither Aboriginal nor Torres Strait Islander	1.00	1.00	1.00
Country of birth	Australia	1.00	1.00	1.00
	Overseas	1.27 [0.95, 1.71]	0.94 [0.60, 1.46]	0.47 [0.18, 1.19]
Sexual orientation	Heterosexual	1.00	1.00	1.00
	Gay or lesbian/bisexual/different term	**1.93 [1.26, 2.95]**	**2.07 [1.10, 3.93]**	**2.91 [1.09, 7.74]**
Employment	Employed	1.00	1.00	1.00
	Unemployed	1.75 [0.83, 3.69]	1.01 [0.32, 3.26]	1.09 [0.24, 5.0]
	Not in the labour force	**1.59 [1.08, 2.33]**	1.19 [0.73, 1.96]	1.00 [0.47, 2.11]
Remoteness area (ARIA)	Major cities	1.00	1.00	1.00
	Inner regional	0.95 [0.67, 1.37]	1.28 [0.78, 2.09]	1.05 [0.31, 3.56]
	Outer regional/remote /very remote	0.99 [0.47, 2.08]	1.00 [0.45, 2.21]	1.69 [0.41, 6.93]
Index of Relative Socio-economic Disadvantage (IRSD)	1 (most disadvantaged)	1.45 [0.91, 2.32]	1.46 [0.74, 2.89]	**5.18 [1.18, 22.78]**
	2	1.08 [0.68, 1.70]	1.26 [0.64, 2.46]	3.99 [0.70, 22.68]
	3	1.09 [0.73, 1.62]	1.58 [0.81, 3.05]	2.17 [0.29, 16.32]
	4	1.07 [0.67, 1.71]	0.90 [0.48, 1.70]	1.84 [0.35, 9.61]
	5 (least disadvantaged)	1.00	1.00	1.00
Any 12-month affective disorder	Yes	**4.87 [3.62, 6.57]**	**6.05 [3.63, 10.09]**	**5.04 [1.68, 15.08]**
	No	1.00	1.00	1.00
Any 12-month anxiety disorder	Yes	**3.67 [2.52, 5.33]**	**3.62 [2.09, 6.26]**	**8.82 [2.19, 35.50]**
	No	1.00	1.00	1.00
Any 12-month substance use disorder	Yes	**1.95 [1.22, 3.13]**	**2.29 [1.30, 4.03]**	**4.22 [1.88, 9.51]**
	No	1.00	1.00	1.00

aOR: adjusted odds ratio; 95% CI: 95% confidence interval. The point estimates highlighted in bold are statistically significant in Table 3.

Table 4 shows the health services that were used by participants who made a suicide attempt in the previous 12 months in the immediate aftermath of the attempt. Nearly two-fifths of participants did not use health services. Around one-third of participants used crisis support and counselling services, hospital emergency departments, psychiatrists or psychologists, and general practitioners (GPs). Around one-quarter used emergency response services and hospital inpatient services. In each case, the majority of participants who used a given service felt that they received as much help as they needed from that service. There was particularly strong endorsement for hospital inpatient services, with 91.8% of participants who were admitted to hospital following their suicide attempts indicating that they received sufficient help in this setting.

**Table 4.** Type of health service received immediately following suicide attempt in the last 12 months, 2020–2022.

	Used service		Received as much help as needed from service	
	%^a^	95% CI	%^b^	95% CI
Crisis support and counselling service	36.0	[20.5, 55.0]	N/A	N/A
Emergency response services (e.g. police, ambulance officer or paramedic)	26.1	[14.6, 42.4]	75.3	[48.0, 90.9]
Hospital emergency department	35.8	[21.2, 53.7]	81.6	[48.0, 95.5]
Hospital inpatient service (admitted to hospital for one or more nights)	24.6	[13.0, 41.6]	91.8	[62.9, 98.7]
General practitioner	32.3	[17.6, 51.6]	74.0	[41.6, 91.9]
Psychiatrist or psychologist	35.7	[21.6, 52.7]	68.7	[45.0, 85.5]
Lived experience worker or peer worker	N/A	N/A	0	0
Other health professional or peer worker	N/A	N/A	N/A	N/A
Did not use health service	37.8	[21.8, 57.1]	Not applicable	Not applicable

95% CI: 95% confidence interval.

N/A indicates that data are not available for publication due to small cell counts.

a Of those who made a suicide attempt in the last 12 months.

b Of those who used the given service.

Table 5 shows the association between suicide attempts and self-harm without suicidal intent for the 2020–2022 survey participants. In the 12 months before the survey, 0.1% of participants attempted suicide only, 1.6% self-harmed without suicidal intent only and 0.2% attempted suicide and self-harmed. It is worth considering these figures in the light of the fact that, in total, 0.3% of participants attempted suicide (Table 1). This means that two-thirds of those who attempted suicide during the previous year also self-harmed without suicidal intent at some point in the same period.

**Table 5.** 12-month rates of suicide attempts and self-harm without suicidal intent, all combinations, 2020–2022.

	Persons	
	%	95% CI
12-month suicide attempt only	0.1	[0.1, 0.2]
12-month self-harm only	1.6	[1.3, 1.9]
Both 12-month suicide attempt and 12-month self-harm	0.2	[0.1, 0.3]
Neither 12-month suicide attempt nor 12-month self-harm	98.1	[97.8, 98.4]

95% CI: 95% confidence interval.

## Discussion

### Summary and interpretation of findings

Our study presents updated lifetime and annual rates of suicidal thoughts and behaviours among the Australian adult population, using data from the 2020–2022 NSMHWB. Over their lifetime, one in six adults had seriously considered suicide, one in 13 had made a suicide plan and one in 20 had made a suicide attempt. Over a 12-month period, one in 30 adults had seriously considered suicide, one in 91 had made a suicide plan and one in 333 had made a suicide attempt. Comparisons with data from the 2007 survey suggest that the situation is worse than it was 15 years ago in terms of suicidal ideation and suicide plans (although not suicide attempts), with notable increases for males and for those aged 16–24 and 45+. Young people are of particular concern because they had elevated rates in 2007, and their 2020–2022 rates have doubled in the interim.

Another way of looking at this is to consider the proportion of people who experience suicidal ideation who then go on to make a suicide plan or attempt suicide. The skip logic in the 2020–2022 survey made this possible; as noted, only those participants who endorsed having suicidal ideation in the past 12 months were asked about whether they had made a suicide plan or attempted suicide in same period. The same skip logic was used in 2007. It is therefore possible to determine the proportion of people who thought about suicide who then went on to make a suicide plan or attempt for each survey. In 2020–2022, around one-third of those who seriously thought about suicide made a suicide plan and around one-tenth attempted suicide. In 2007, a lower proportion made a suicide plan (around one-quarter), but a higher proportion attempted suicide (around one-fifth).

Some of the observed patterns of suicidal thoughts and behaviours may be related to the COVID-19 pandemic and its associated stresses. In Australia, public health responses to the pandemic (e.g. lockdowns) began in March 2020 and largely ceased in December 2021, so all participants in the first cohort and some in the second would have completed the survey during the pandemic. Our finding that 12-month rates of suicidal ideation and suicide plans increased between 2007 and 2020–2022 but 12-month rates of suicide attempts remained the same corresponds with other studies. Studies that have pooled data from multiple countries have found rates of suicidal ideation during the pandemic to be higher than pre-pandemic rates (Farooq et al., 2021), but have not consistently demonstrated increases in rates of hospital presentations for suicide attempts (Steeg et al., 2022) or suicide (Pirkis et al., 2021, 2022a). Findings from Australian studies support this picture. One longitudinal study found that community rates of suicidal ideation were high (albeit steady) during the pandemic (Batterham et al., 2022), and another study identified an increase in hospital presentations for suicidal ideation but not for suicide attempts (Sperandei et al., 2022).

Having said this, the pandemic is unlikely to be the only explanation for the observed increases in suicidal ideation and suicide plans. In the case of young people, e.g., we know that mental health issues and psychological distress have risen in the past 20 years (Botha et al., 2023; Enticott et al., 2022). Various factors have been implicated, including concerns about climate change and financial hardship (Elgar et al., 2015; Walsh et al., 2021), social media’s adverse impacts (Schønning et al., 2020) and insufficient access to services (Barican et al., 2022). It is plausible that the influence of these and other factors has contributed to increased rates of suicidal ideation and suicide plans.

Our study also indicated that in 2020–2022 certain socio-demographic and clinical factors were associated with survey participants experiencing suicidal thoughts and behaviours in the previous year. Again, males and young people featured in this finding; males were more likely than females to make a suicide plan, and those aged 16–24 were more likely than older people to make a suicide attempt. People who identified as gay, lesbian, or bisexual or used some other term to describe their sexual identity were more likely to consider suicide, make a suicide plan and make a suicide attempt than those who identified as heterosexual. Socio-economic factors were also significant, with people outside the labour force being more likely to experience suicidal ideation than employed people, and people living in the most disadvantaged areas being more likely to attempt suicide than those from more affluent areas. People with affective disorders, anxiety disorders and/or substance use disorders were more likely to experience suicidal ideation, make a suicide plan and attempt suicide than those without these disorders. These findings are consistent with those from other studies. For example, previous studies have highlighted higher risk of suicidal thoughts and behaviours among sexual minority populations (King et al., 2008; Marshal et al., 2011). Similarly, mental disorders – particularly affective disorders – have been shown to be associated with suicidal thoughts and behaviours in previous studies (Nock et al., 2010), including those using data from the 1997 and 2007 NSMHWB (Johnston et al., 2009; Pirkis et al., 2000).

Our study showed that around three-fifths of those who made a suicide attempt in the previous 12 months used services, with counselling and support service, hospital emergency departments, psychiatrists or psychologists, and GPs being particularly common sources of help. In the main, participants were positive about the services they used, with the majority indicating that they received as much help as they needed. However, it is concerning that two-fifths of participants who made a suicide attempt did not use any services. Some may not have felt they had any need to do so, but others may have struck barriers relating to lack of availability of services, previous poor experiences with services, or stigma (Hom et al., 2015). Further work is needed to ensure that anyone who makes a suicide attempt can access appropriate services and receive care that benefits them.

Ours is one of the first population-based studies to consider suicide attempts and self-harm without suicidal intent simultaneously. There is debate in the literature about the distinction between these behaviours (Huang et al., 2020), and our study contributes to this debate by showing that two-thirds of those who make a suicide attempt also self-harm without suicidal intent. They may do so for emotional relief, or as a means of coping with distressing thoughts or feelings, or for other reasons (Cooper et al., 2011). In many instances, the motives underlying acts of self-harm will not be clear. Individuals themselves will not always be able to articulate why they self-harmed, and on any given occasion, they may have multiple reasons for doing so (Cooper et al., 2011). Our findings suggest that trying to distinguish between those who make a suicide attempt and those who self-harm for other reasons may create a false dichotomy.

### Implications for policy

Our findings underscore the need for the new National Suicide Prevention Strategy to consider the full spectrum of suicidal thoughts and behaviours, not just suicide. The numbers of people who experience these thoughts and behaviours are unacceptably high, and substantial proportions of those who think about suicide progress to planning or attempting to take their own lives. Policy efforts to date have not reduced these suicidal thoughts and behaviours, and in fact, suicidal ideation and suicide plans have continued to rise. We would go one step further and suggest that the new Strategy should also consider self-harm more broadly, given the significant overlap between suicide attempts and self-harm without suicidal intent. Interventions designed to address suicidal ideation, suicide plans, suicide attempts and self-harm without suicidal intent may ultimately bring down the suicide rate.

In addition, our findings provide support for the new Strategy taking a public health approach to preventing suicidal thoughts and behaviours. This approach was outlined in the blueprint paper that underpins the Strategy (Pirkis et al., 2022b, 2023) and would see the Strategy addressing key risk factors for suicidal thoughts and behaviours. Our study points to the fact that these should include, but not be limited to, clinical factors like mental disorders; the Strategy should also address the needs of priority groups like young people, people from sexual minority groups and people facing socio-economic disadvantage.

### Study limitations

Some participants may not have felt comfortable self-reporting their suicidal thoughts and behaviours because of the stigma associated with them (Carpiniello and Pinna, 2017; Mérelle et al., 2018). The fact that participants who did not report suicidal ideation were not asked about suicide plans or suicide attempts meant that impulsive suicide attempts may not have been captured.

The relative rarity of suicidal thoughts and behaviours and the sample size meant that we were unable to conduct any sub-analyses apart from calculating rates for broad sex and age groups, due to small numbers. For example, we were unable to explore associations between specific mental health disorders (e.g. bipolar disorder, alcohol use disorder) and suicidal thoughts and behaviours due to the small numbers of people living with both. Small numbers also restricted our ability to report suicidality estimates for minority population groups such as Indigenous people and LGBTQIA+ people. These small-number issues were particularly evident for those who made suicide attempts and those who self-harmed without suicidal intent. Low numbers also affected the degree of uncertainty around our estimates.

We did not have any information on non-responders, and there may have been systematic differences between them and those who chose to participate.

## Conclusion

Our study has shown that rates of suicidal ideation and suicide plans increased over the last 15 years. Rates of suicide attempts did not change. Suicidal thoughts and behaviours confer risk for suicide and are significant problems in their own right. The new National Suicide Prevention Strategy will need to promote a strong whole-of-government approach that actively targets groups at heightened risk.
